# Fluorescent property of 3-hydroxymethyl imidazo[1,2-a]pyridine and pyrimidine derivatives

**DOI:** 10.1186/1752-153X-6-83

**Published:** 2012-08-07

**Authors:** Stephania Velázquez-Olvera, Héctor Salgado-Zamora, Manuel Velázquez-Ponce, Elena Campos-Aldrete, Alicia Reyes-Arellano, Cuauhtémoc Pérez-González

**Affiliations:** 1 Departamento Química Orgánica. Escuela Nacional Ciencias Biológicas, Instituto Politécnico Nacional, México City,, Zip code 11340, , Mexico; 2 Departamento de formación básica disciplinaria Instituto Politécnico Nacional. Mineral de Valenciana No 200 Fraccionamiento Industrial Puerto Interior 36275, Silao de la Victoria, Guanajuato, Mexico; 3 Departamento Química Orgánica. Escuela Nacional Ciencias Biológicas, Instituto Politécnico Nacional, México City, Zip code 11340, Mexico; 4 Departamento de Sistemas Biológicos. Universidad Autónoma Metropolitana Xochimilco, Mexico City, Zip code 23-181, Mexico

## Abstract

**Background:**

Imidazo[1,2-a]pyridines and pyrimidines are important organic fluorophores which have been investigated as biomarkers and photochemical sensors. The effect on the luminescent property by substituents in the heterocycle and phenyl rings, have been studied as well. In this investigation, series of 3-hydroxymethyl imidazo[1,2-a]pyridines and pyrimidines were synthesized and evaluated in relation to fluorescence emission, based upon the hypothesis that the hydroxymethyl group may act as an enhancer of fluorescence intensity.

**Results:**

Compounds of both series emitted light in organic solvents dilutions as well as in acidic and alkaline media. Quantitative fluorescence spectroscopy determined that both fused heterocycles fluoresced more intensely than the parent unsubstituted imidazo[1,2-a]azine fluorophore. In particular, 3-hydroxymethyl imidazo[1,2-a]pyridines fluoresced more intensely than 3-hydroxymethyl imidazo[1,2-a]pyrimidines, the latter emitting blue light at longer wavelengths, whereas the former emitted purple light.

**Conclusion:**

It was concluded that in most cases the hydroxymethyl moiety did act as an enhancer of the fluorescence intensity, however, a comparison made with the fluorescence emitted by 2-aryl imidazo[1,2-a]azines revealed that in some cases the hydroxymethyl substituent decreased the fluorescence intensity.

## Background

In the past few years a growing interest in the chemistry of imidazo[1,2-a]pyrimidines and pyridines has been developed due to the extent of their applications in pharmacological science. Indeed they are known for their anxiolytic [1], cardiovascular [2], analgesic [3,4], antihypertensive [4] and neuroleptic [5,6] among other activities [7-9]. However, imidazo[1,2-a]pyridines and pyrimidines are also attractive due to their physicochemical properties exhibited, namely the fluorescent activity. Several studies concerning the effects of substituents on the fluorescent properties of imidazo[1,2-*a*pyridines have been carried out, for instance, 2-phenyl (or 2-(2-naphthyl)) and/or 7-methyl substitution caused no deterioration of the fluorescent property. The amino or dimethylamino substitution at the 4^'^-position of 2-phenyl imidazo[1,2-*a*pyridine shifted the fluorescence to the visible region in polar solvents [10]. From a comparative spectroscopy study performed on several imidazo[1,2-a]pyridines and pyrimidines, it was observed that substitution of a proton for methyl, carboxyl, or amino group increased the fluorescence intensity. Fluorescence was destroyed when a ring position carried a nitro group or when the pyridine or pyrimidine ring is catalytically reduced to the 5,6,7,8-tetrahydro derivative [11]. In the latter study, it was also observed that imidazo[1,2-a]pyrimidines fluoresced more intensely and ca. 60 nm higher than the analogous pyridines. Taking advantage of the fluorescence property, a imidazo1,2-a]pyridine derivative was used as a biomarker of hypoxic tumor cells [12]. A (4-piperidinylfluorophenyl) imidazo1,2-a]pyridine was applied to a multiple fluorescent chemosensor [13]. Recently, an imidazopyrimidine based compound was used in an electron transport layer of an organic light emission device [14].

However, a great disadvantage of many current fluorophores is their very short time of life and susceptibility to physicochemical environments [14], therefore interest in the development of more efficient fluorophores is growing. In this study, the hydroxymethyl group was investigated as an enhancer of the fluorescence property of derivatives of imidazo[1,2-a]pyridines and imidazo[1,2-a]pyrimidines with the aim of obtaining fluorophores with potential use as biomarkers.

## Results and discussion

The hydroxymethyl group has proved to be a promoter of fluorescence in naphthyl thioureas [15]. In another study, it was reported that 2-(3,4,5,6-tetrafluoro-2-hydroxyphenyl)imidazo[1,2-a]pyridine emitted long wavelength light around 540 nm both in polar and in nonpolar solvents [16]. Based upon this information, it was hypothesized that introduction of a hydroxymethyl group at position 3 of the imidazo[1,2-a]azines should enhance fluorescence (probably through a spatial non-covalent interaction of the hydroxyl non-bonding electrons with the aryl rings) as compared to the parent fluorophore (the unsubstituted imidazo[1,2-a]azine).

**Scheme 1:**
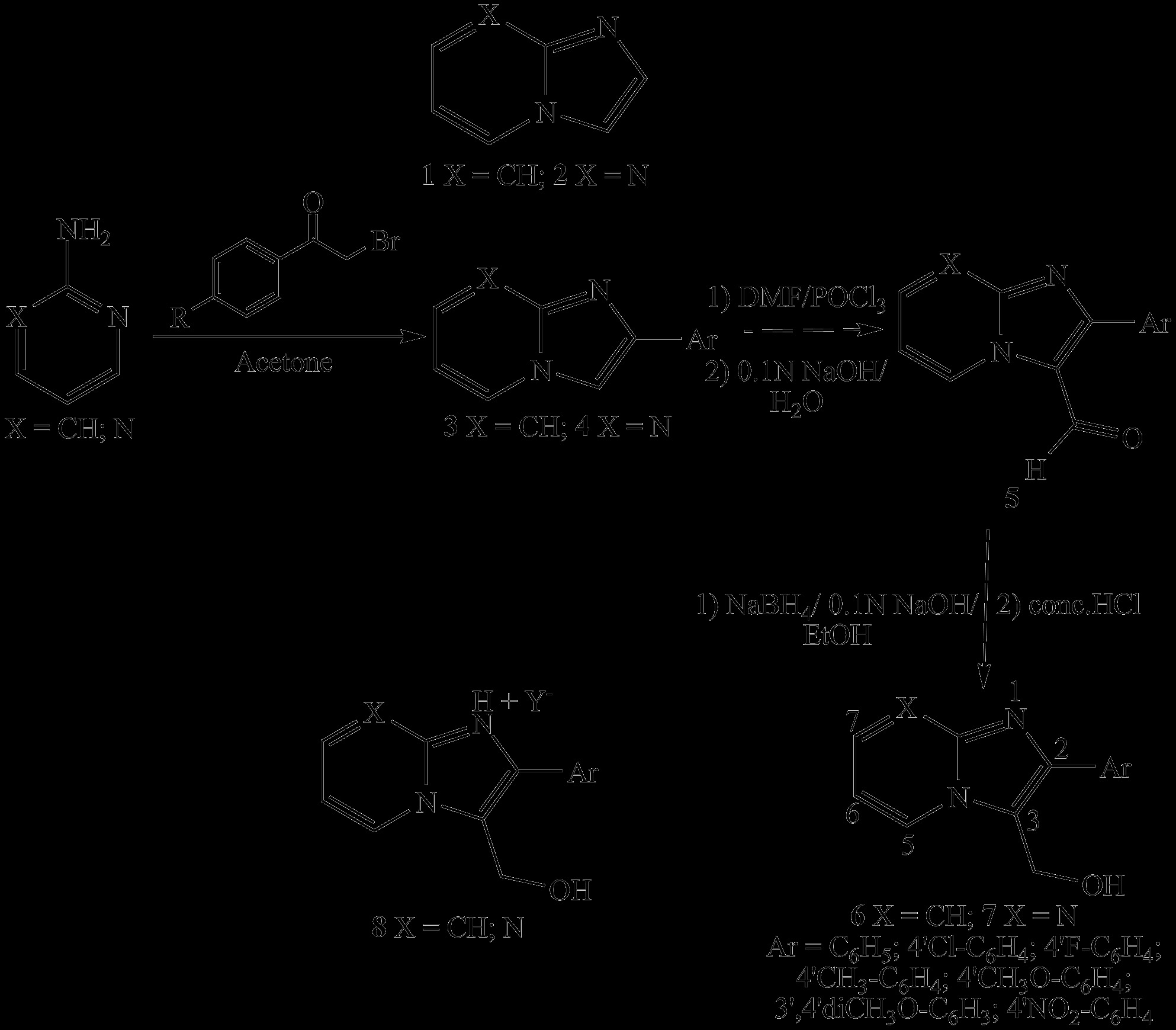
Synthesis of 3-hydroxymethyl imidazo[1,2-a]azines and structures 1, 2 and 8.

The two series of compounds were prepared straightforward (Scheme 1). Condensation of 2-amino pyridine or 2-amino pyrimidine with the appropriately substituted 2-bromo acetophenone afforded the imidazo[1,2-a]azine nucleus. A Vilsmeier Haack treatment on the fused heterocycles 3 and 4 led to the corresponding 3-formyl substituted derivatives 5. Reduction of the formyl moiety with NaBH_4_ in alkaline ethyl alcohol delivered the 3-hydroxymethyl derivatives 6 and 7.

Some of these products are already known but have not been studied in relation to a fluorescence property. Products were fully identified by spectroscopic methods and were obtained in moderate to good yields as shown in Tables 1 and 2. New compounds were submitted to combustion analysis for complete characterization.

**Table 1:** Yields and melting points of 2-aryl-3-hydroxymethyl imidazo[1,2-a]pyridines 6

**Compound 6**	**Aryl**	**Yield (%)**	**Mp (°C) Experimental**	Mp (°C) Literature*
**a**	C_6_H_5_	70%	202 - 203	>212
**b**	4’Cl-C_6_H_4_	61.3%	229 - 230	>240
**c**	4’F-C_6_H_4_	70.3%	154 - 155	153 - 155
**d**	4’CH_3_-C_6_H_4_	76.7%	274 - 275	>290
**e**	4’CH_3_O-C_6_H_4_	66.7%	166 - 167	165 - 167
**f**	3’,4’di-CH_3_O-C_6_H_3_	71%	171 - 172	170 - 172
**g**	4’NO_2_-C_6_H_4_	70%	225 - 226	224 – 226

* Reference [17].

**Table 2:** Yields and melting points of 2-aryl-3-hydroxymethyl imidazo[1,2-a]pyrimidines 7

**Compound 7**	**Aryl**	**Yield (%)**	**Mp (°C)**
**a**	C_6_H_5_	60.6%	241 – 242*
**b**	4’Cl-C_6_H_4_	63.8%	164 - 165
**c**	4’F-C_6_H_4_	30.3%	232 - 233
**d**	4’CH_3_-C_6_H_4_	32%	199 - 200
**e**	4’CH_3_O-C_6_H_4_	86%	187 - 188
**f**	3’,4’di-CH_3_O-C_6_H_3_	30.4%	200 - 201
**g**	4’NO_2_-C_6_H_4_	72%	213 - 214

*Compound 2a is mentioned in reference [18], mp is not given.

A series of qualitative assays aimed to evaluate the stability of the products and permanence of the fluorescent property followed: Compound 6 g did not fluoresce. Dilutions of the compounds (0.05 g) in common organic solvents (10 mL) such as ethyl alcohol, acetone, ethyl acetate, acetonitrile and dichloromethane showed fluorescence, this being considerably intense in ethyl acetate and dichloromethane. Solutions of 3-hydroxymethyl imidazo[1,2-a]pyridine 6 and pyrimidine 7 derivatives at various concentrations of aqueous HCl (0.1, 1 and 8 N) preserved the fluorescence, however, this was suppressed with 10 N HCl for the case of imidazo[1,2-a]pyrimidines 7 and deteriorated for the case of 6 at both λ 250 and 365 nm. Addition of weakly acidic materials also preserved fluorescence. Thus, heterogeneous 5% aqueous NH_4_Cl mixtures of 3-hydroxymethyl 6d showed a decreased fluorescence but 7d preserved it. A powdered mixture of silica gel (0.5 g), for column chromatography thoroughly mixed with the alcohol derivative 7d (10 mg) fluoresced only at the long wavelength λ 365 nm whereas 6d (10 mg) fluoresced at both wavelengths. These results indicate that the protonated species 8 holds the fluorescence activity to a certain extent. Fluorescence of both 6d and 7d in solutions 0.1, 1 and 10 N NaOH gradually diminished but prevailed at both wavelenghts. Biological materials such as egg yolk, pig blood, albumina and *Giardia lamblia* cultures were fluorescent upon addition of the imidazo[1,2-a]azines. The fluorescence of these products remained after several days.

The quantitative fluorescence analysis performed on one hundred-fold dilutions of the compounds showed that imidazo[1,2-a]pyridines absorbed and emitted energy at long wavelengths (Table 3) while imidazo[1,2-a]pyrimidines absorbed at lower wavelengths but emitted light at longer wavelengths (Table 4).

**Table 3:** Spectroscopic data for 2-aryl-3-hydroxymethyl imidazo[1,2-a]pyridines 6

		**UV / Vis**		**Fluorescence**	
**Compound**	**Aryl**	**Abs**_**λ max**_**(nm)**	**λ ex (nm)**	**λ em (nm)**	I*
**1**	---	318	320	376	101.9
**6a**	C_6_H_5_	320	320	381.6	346.91
**6b**	4’Cl-C_6_H_4_	323	320	381	147.17
**6c**	4’F-C_6_H_4_	320	320	380.4	340.42
**6d**	4’CH_3_-C_6_H_4_	323	320	381.2	306.31
**6e**	4’CH_3_O-C_6_H_4_	323	320	383.6	884.37
**6f**	3’,4’di- CH_3_O-C_6_H_3_	323	320	385.6	878.18
**6g**	4’NO_2_-C_6_H_4_	347	347	-	-

*I Fluorescence intensities were standardized to a Raman blank methanol solution.

**Table 4:** Spectroscopic data for 2-aryl-3-hydroxymethyl imidazo[1,2-a]pyrimidines 7

		**UV / Vis**			**Fluorescence**
**Compound**	**Aryl**	**Abs**_**λ max**_**(nm)**	**λ ex (nm)**	**λ em (nm)**	I*
**2**	---	229	230	414.8	205.58
**7a**	C_6_H_5_	247	247	437.6	432.44
**7b**	4’Cl-C_6_H_4_	228	228	439.2	53.56
**7c**	4’F-C_6_H_4_	240	240	437.2	542.19
**7d**	4’CH_3_-C_6_H_4_	240	240	433.6	184.23
**7e**	4’CH_3_O-C_6_H_4_	226	226	433.6	188.03
**7f**	3’,4’di-CH_3_O-C_6_H_3_	344	344	432.4	428.32
**7g**	4’NO_2_-C_6_H_4_	265	265	432	186.43

*I Fluorescence intensities were standardized to a Raman blank methanol solution.

In the case of the imidazo[1,2-a]pyridines 6, all compounds fluoresced more intensely than the parent unsubstituted imidazo[1,2-a]pyridine 1, with the exception of the nitro substituted 6 g, which showed no fluorescence as was expected. It is also worth noting that the 2-(4-chlorophenyl) substituted imidazopyridine derivative 6b showed a rather low intensity.

As for the case of the imidazo[1,2-a]pyrimidine series, again the 2-(4’-chlorophenyl) substituted imidazopyrimidine 7b showed a very low intensity. Surprisingly, the electron donor substituted 7d and 7e and the nitro substituted derivative 7 g registered a similar intensity close to that given by the parent fluorophore 2.

**Figure 1:**
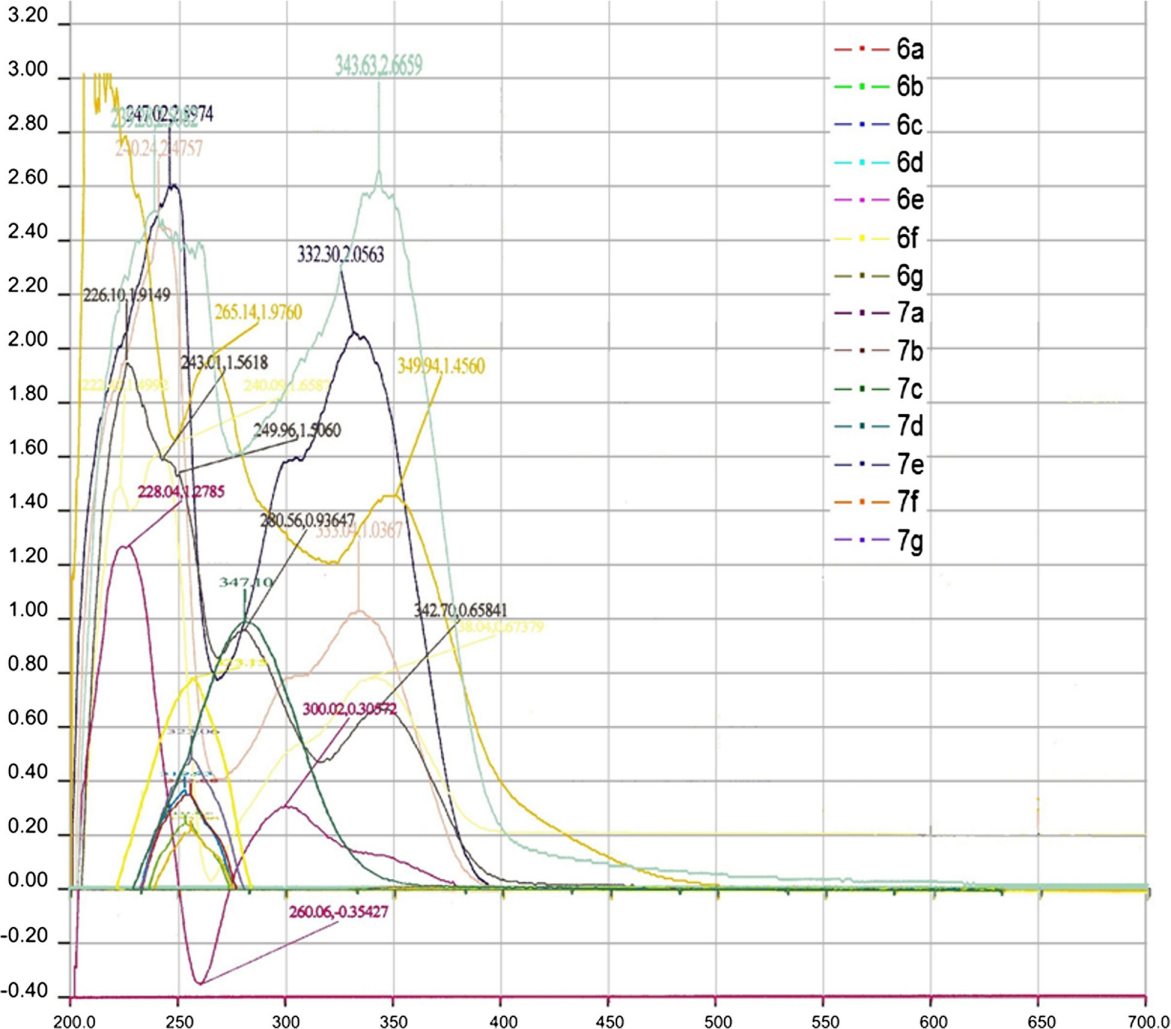
UV-Visible absorption spectra of imidazo[1,2-a]azines 6 and 7.

A comparative UV absorption graph for both series of compounds is shown in Figure 1.

**Figure 2:**
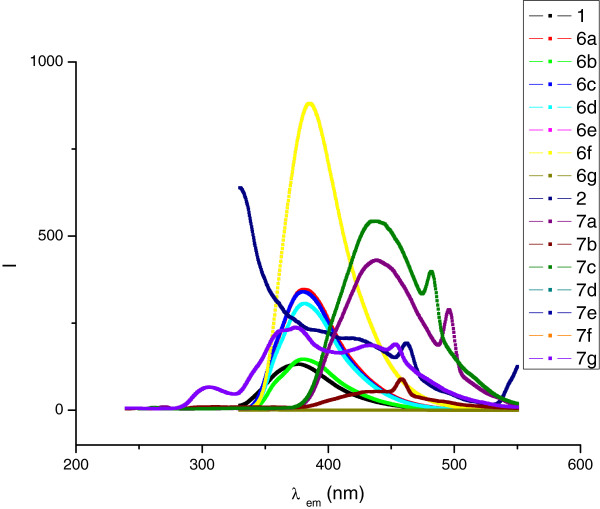
A comparison of emission wavelengths and intensity of imidazo[1,2-a]azines 6 and 7.

Figure 2 shows a comparison of the emission intensities. From these, it is clearly appreciated that imidazo[1,2-a]pyrimidines emitted light at longer wavelengths than analogous imidazo[1,2-a]pyridines but the latter fluoresced more intensely, in contrast to previous analyses on imidazo[1,2-a[azines [12]. The effects of the phenyl substituents on the outcome of the emission intensities did not follow a consistent pattern, some interesting results are worth pointing out. The strong electron-donating methoxy group caused a marked increased intensity on the imidazopyridine (6e) but not on the imidazopyrimidine (7e). The fluorescence intensity of the imidazo[1,2-a]azines with the phenyl carrying the electron-withdrawing chlorine was low in 6b and dramatically decreased in 7b, whereas the intensity of the derivative with the phenyl bearing the fluorine substituent, 7c was enhanced to make it the most fluorescent of the imidazopyrimidine series. Interestingly in the case of the 4’-nitrophenyl substituted imidazo[1,2-a]pyridine, fluorescence was completely absent, while in the 4’-nitrophenyl substituted imidazo[1,2-a]pyrimidine fluorescence did not vanish.

According to the previous results, the role of the hydroxymethyl moiety was not quite clear, therefore we decided to revise the influence of the aryl group on the luminescent response of the 2-aryl imidazo[1,2-a]azines. Results for the 2-aryl imidazo[1,2-a]pyridines 3 (Table 5) showed that the phenyl, as well as the 4’-chloro, 4’-fluoro and 4’-methoxy substituted phenyl increased the fluorescence intensity as compared to 1 and interestingly the 3,4-dimethoxyphenyl caused a drop-off of the intensity. In most cases, the hydroxymethyl group increased fluorescence intensity even more than the aryl substituent, therefore it acted as enhancer. However, the intensity was decreased in the 4’-fluoro substituted phenyl and even more in the case of the 4’-chloro substituted phenyl.

**Table 5:** Fluorescence data for 2-aryl imidazo[1,2-a]pyridines 3

	**R**	**UV / Vis**		**Fluorescence**	
**Compound**	**Aryl**	**Abs**_**λ max**_**(nm)**	**λ ex (nm)**	**λ em (nm)**	I*
1	---	318	320	376.0	101-9
3a	C_6_H_5_	237	240	375.6	367.8
3b	4’Cl-C_6_H_4_	245	245	375.2	310.92
3c	4’F-C_6_H_4_	300	300	374	470.34
3e	4’MeO-C_6_H_4_	315	315	381.6	407.83
3f	3’,4’diMeO- C_6_H_3_	228	230	385.6	178.19

*I Fluorescence intensities were standardized to a Raman blank methanol solution.

**Figure 3:**
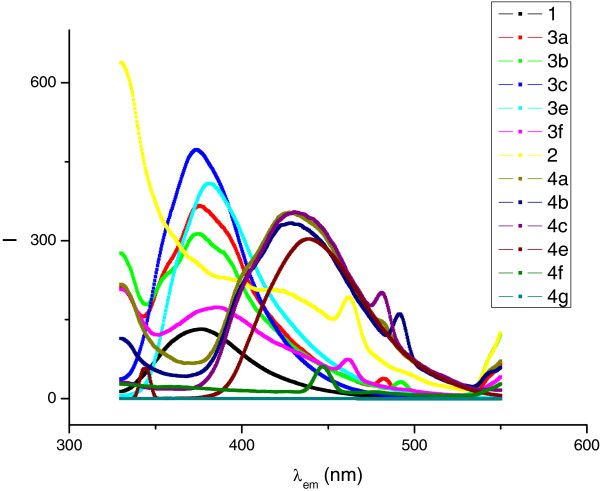
A comparison of emission wavelengths and fluorescence intensity for imidazo[1,2-a]pyridines 3 and imidazo[1,2-a]pyrimidines 4.

As for the case of imdazopyrimidines (Table 6), substitution by phenyl did increase the intensity as compared to the parent fluorophore 2, the 4’-nitro substituted phenyl did not show fluorescence and it was interesting to observe that the 4’-methoxy phenyl (4f) showed a very low intensity. With this information, it was concluded that the hydroxymethyl moiety was a promoter of fluorescence in compound (4 g) and acted as an enhancer of the intensity in all other derivatives except in 2-(4’-chlorophenyl) imidazo[1,2-a]pyrimidine where a decrement was found. Figure 3 shows a comparative graph of the emission wavelengths and fluorescence obtained for compounds 3 and 4.

**Table 6:** Fluorescence data for 2-aryl imidazo[1,2-a]pyrimidines 4

		**UV / Vis**		**Fluorescence**	
**Compound**	**Aryl**	**Abs**_**λ max**_**(nm)**	**λ ex (nm)**	**λ em (nm)**	**I**
2	---	229	230	414.8	205.58
4a	C_6_H_5_	237	240	429.2	351.98
4b	4’Cl-C_6_H_4_	245	245	428.4	334.1
4c	4’F-C_6_H_4_	240	240	431.6	355.16
4e	4’MeO-C_6_H_4_	240	240	439.2	303.01
4f	3’,4’diMeO-C_6_H_3_	222	222	447.6	55.998
4g	4’NO_2_-C_6_H_4_	352	352	---	---

*I Fluorescence intensities were standardized to a Raman blank methanol solution.

### Thermal properties

**Figure 4:**
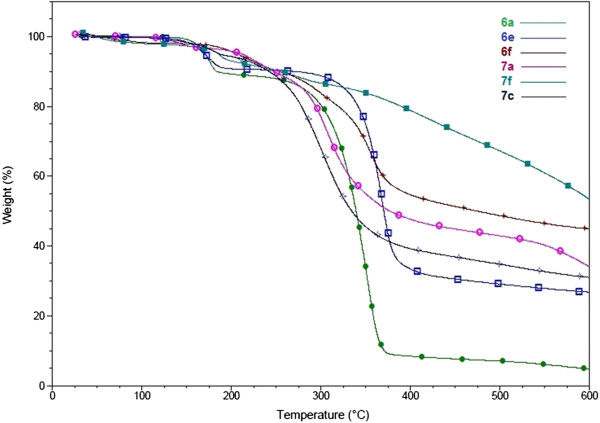
Thermogravimetric analysis of selected 3-hydroxymethyl imidazo[1,2-a]pyridine and pyrimidine derivatives.

The thermal stability of the most fluorescent compounds, 6a, 6e, 6f, 7a, 7c and 7f was estimated through a thermo gravimetric analysis (TGA) within an interval 0 – 600°C under an inert atmosphere. From the TG curves shown in Figure 4 it was determined that these compounds were thermally stable up to 280°C. The thermodynamic most stable was 7f, which completely decomposed above 600°C (47.75% mass loss at 600°C). The chemically most stable was 6f (55.25% weight loss at 600°C) and compound 7a showed a 66.47% weight loss at 600°C, the least stable was 6a (95.25% weight loss at 600°C).

### Experimental

Melting points were measured on an Electrothermal melting point apparatus and are uncorrected. The FT-IR spectra were recorded on Perkins-Elmer 257 spectrometer using KBr discs. ^1^ H and ^13^C nmr spectral data were recorded with a Varian Mercury 300 MHz or a Varian 500 spectrometer. The chemical shifts (δ) are referenced to internal (CH_3_)_4_Si (δ ^1^ H = 0, δ ^13^C = 0). Fluorescence measurements were performed with a Shimadzu spectrofluorophotometer (RF-5000), equipped with a 150-W Xenon lamp, 12” color video display, 1 x 1 cm quartz cells. Both, operational performance and instrument sensitivity were revised by running a Raman spectrum of methanol. Cells were mounted in the holding device. The cell was filled out with a dilution 1:100 of the compounds in question for various lengths and then washed four times with methanol. Thermo gravimetric analysis was performed on a TGA 2950 Thermogravimetric Analyzer TA Instruments in a range 0-600°C, at a rate of 10°C/min on a platinum tray, under a nitrogen atmosphere and a flux rate 37.5 and 25.

### General procedure for the synthesis of 3-hydroxymethyl imidazo[1,2-a]pyridines and pyrimidines

In a round bottom flask equipped with a magnetic stirrer, 1 g of the formyl derivative was suspended in ethanol (30 mL). The mixture was heated to 30°C and NaBH_4_ dissolved in 2 mL of 0.1 N NaOH added (2 molar equivalents for imidazo[1,2-*a*]pyridine and 1 molar equivalent for imidazo[1,2-*a*]pyrimidine). The reaction mixture was left stirring and reaction progress monitored by thin layer chromatography. After completion, ethanol was removed under vacuum. The solid formed was re-suspended in water and concentrated HCl added dropwise to neutral pH. The solid was collected by filtration, washed with water and dried off with hexane.

#### 2-Phenyl-3-hydroxymethy imidazo[1,2-a]pyridine 6a

IR υ max cm^-1^: 3290 (OH); 2966.57, 2916.51, 2848.34 (C-H); 1734.11 (C=C).

^1^H NMR (300MHz, DMSO-d_6_) δ: 8.46 (1H, dd, *J*_5,6_=6.9, *J*_5,7_=1.2, H_5_); 7.84 (2H, d, *J*_2’,3’_=6.9, H_2’,6’_); 7.61 (1H, dd, *J*_8,7_=8.1, *J*_8,6_=1.2, H_8_); 7.48 (2H, td, *J*_3’,2’_=6.9, *J*_3’,4’_=7.2, *J*_3’,5’_=1.6 H_3’,_H_5’_); 7.38 (1H, td, *J*_4’,3’_=7.2, *J*_4’,2’_=1.6 H_4’_); 7.3 (1H, td, *J*_7,8_=8.1, *J*_7,6_=6.9, *J*_7,5_=1.2, H_7_); 6.98 (1H, td, *J*_6,5_=6.9, *J*_6,7_=6.9, *J*_6,8_=1.2, H_6_); 5.44 (1H, s, OH); 4.92 (2H, s, CH_2_).

^13^C NMR (75.5MHz, DMSO-d_6_) δ: 143.9 (C_5_), 142.8 (C_8a_); 134.3 (C_1’_), 128.4 (C_2’_); 128.1 (C_3’_); 128.2 (C_4’_); 124.8 (C_7_); 120.4 (C_3_); 116.6 (C_8_); 111.9 (C_6_); 52.8 (CH_2_).

#### 2-(4’-Chlorophenyl)-3-hydroxymethyl imidazo[1,2-a]pyridine 6b

Isolated as a white solid in 61.3% yield, mp 229 - 230ºC (Lit 15, mp >240°C).

IR υ max cm^-1^: 3398 (OH); 2930 (C-H); 1635 (C=C).

^1^H NMR (300MHz, DMSO-d_6_) δ: 8.46 (1H, d, *J*_5,6_=6.9, H_5_); 7.87 (2H, d, *J*_2’,3’_=8.8, H_2’_); 7.6 (1H, d, *J*_8,7_=9.0, H_8_); 7.52 (2H, d, *J*_3’,2’_=8.8, H_3’,5’_); 7.31 (1H, td, *J*_7,8_=9.0, *J*_7,6_=6.8, *J*_7,5_=1.2, H_7_); 6.98 (1H, td, *J*_6,5_=6.9, *J*_6,7_=6.8, *J*_6,8_=1.2, H_6_); 5.47 (1H, t, *J*=4.8, OH); 4.92 (2H, d, *J*=4.8, CH_2_).

^13^C NMR (125MHz, DMSO-d_6_) δ: 143.9 (C_5_); 142.8 (C_8a_); 134.3 (C_1’_); 128.4 (C_2’_); 128.1 (C_3’_); 128.2 (C_4’_); 125.0 (C_2_); 124.8 (C_7_); 120.4 (C_3_); 116.6 (C_8_); 111.9 (C_6_); 52.8 (CH_2_).

#### 2-(4’-Fluorophenyl)-3-hydroxymethyl imidazo[1,2-a]pyridine 6c

Isolated as a white solid in 70.3% yield, mp 154 - 155ºC (Lit 15, mp 153 - 155°C).

IR υ max cm^-1^: 3427 (OH); 3137 and 3048 (C-H); 1600 (C=C).

^1^H NMR (300MHz, DMSO-d_6_) δ: 8.52 (1H, d, *J*_5,6_=6.9, H_5_); 8.0 (2H, dd, *J*_2’,3’_=9.0, *J*_m_=5.7, H_2’_ and H_6’_); 7.57 (1H, d, *J*_8,7_=9.3, H_8_); 7.37 – 7.29 (3H, m, H_7,3’,5’_); 6.87 (1H, d, *J*_6,5_=6.9, H_6_); 5.54 (1H, s, OH); 4.91 (2H, s, CH_2_).

^13^C NMR (125MHz, DMSO-d_6_) δ: 163.4 and 161.0 (C_4_); 143.91 (C_8a_); 130.9 and 130.8 (C_2_); 125.1 (C_1_); 120.4 (C_3_); 116.6 (C_8_); 115.6 and 115.3 (C_3_); 112.1 (C_6_); 52.0 (CH_2_).

#### 2-(4’-Methylphenyl)-3-hydroxymethyl imidazo[1,2-a]pyridine 6d

Isolated as a white solid in 76.7% yield, mp 274 - 275ºC (Lit 15, mp >290°C).

IR υ max cm^-1^: 3279 (OH); 3040 y aprox 2950 (C-H); 1654 (C=C).

^1^H NMR (500MHz, DMSO-d_6_) δ: 8.45 (1H, d, *J*_5,6_=6.9, H_5_); 7.74 (2H, d, *J*_2’,3’_=9.0, H_2’,6’_); 7.61 (1H, d, *J*_8,7_=9.3, H_8_); 7.33 – 7.28 (3H, m, H_7,3’.5’_); 6.98 (1H, dd, *J*_6,5_=6.9, *J*_6,7_=6.9, H_6_); 5.45 (1H, t, *J*=5.1, OH); 4.91 (2H, d, *J*=5.1, CH_2_); 2.36 (3H, s, CH_3_).

^13^C NMR (125MHz, DMSO-d_6_) δ: 143.9 (C_5_); 142.9 (C_8a_); 136.9 (C_1’_); 131.6 (C_4’_); 129.1 (C_3’_); 128.1 (C_2’_); 125.1 (C_2_); 124.9 (C_7_); 120.2 (C_3_); 116.5 (C_8_); 112.0 (C_6_); 52.2 (CH_2_); 20.8 (CH_3_).

#### 2-(4’-Methoxyphenyl)-3-hydroxymethyl imidazo[1,2-a]pyridine 6e

Isolated as a white solid in 66.7% yield, mp 166 - 167ºC (Lit 15, mp 165 - 167°C).

IR υ max cm^-1^: 3427 (OH); ca. 2950 (C-H); 1610 (C=C).

^1^H NMR (300MHz, DMSO-d_6_) δ: 8.43 (1H, dd, *J*_5,6_=6.9, *J*_5,7_=1.5 H_5_); 7.77 (2H, d, *J*_2’,3’_=9.0, H_2’,6’_); 7.57 (1H, d, *J*_8,7_=9.0, H_8_); 7.28 (1H, td, *J*_7,8_=9.0, *J*_7,6_=6.6, *J*_7,5_=1.5, H_7_); 7.04 (2H, d, *J*_3’,2’_=9.0, H_3’,5’_); 6.95 (1H, d *J*_6,7_=6.6, H_6_); 5.37 (1H, d, *J*=5.1, OH); 4.9 (2H, d, *J*=5.1, CH_2_); 3.82 (3H, s, OCH_3_).

^13^C NMR (75.5MHz, DMSO-d_6_) δ: 157 (C_4’_); 141.9 (C_5_); 140.9 (C_8a_); 127.4 (C_2’_); 124.9 (C_4_); 123 (C_1’_); 122.7 (C_2’_); 117.8 (C_3_); 114.5 (C_8_); 112 (C_3’_); 109.8 (C_6_); 53.2 (CH_2_).

#### 2-(3’,4’-Dimethoxyphenyl)-3-hydroxymethyl imidazo[1,2-a]pyridine 6f

Isolated as a brown solid in 71% yield, mp 171 - 172ºC (Lit 15, mp 170 - 172°C).

IR υ max cm^-1^: 3004.19 (OH); 2933.8 and 2834.93 (C-H); 1633.76 (C=C).

^1^H NMR (500MHz, DMSO-d_6_) δ: 8.46 (1H, d, *J*_5,6_=7.2 H_5_); 8.44 (1H, dd, *J*_8,7_=7.0, *J*_8,6_=1.6, H_7_); 7.61 (1H, d, *J*_2’,6’_=1.2, H_2’_); 7.56 (1H, dd, *J*_6’,5’_=6.4, *J*_6’,2’_=1.2 H_6’_); 7.50 (1H, dd, *J*_7,8_=7.0, *J*_7,5_=1.6 H_7_); 6.9 (1H, d, *J*_5’,6’_=8.0, H_5’_); 6.8(1H, td, *J*_6,5_=7.2, *J*_6,7_=7.2 *J*_6,8_=1.2 H_6_) ; 5.4 (1H, s, OH); 4.9 (2H, s, CH_2_); 3.83 (3H, s, OCH_3_), 3.81, (3H, s, OCH_3_).

^13^C NMR (125MHz, DMSO-d_6_) δ: 148.7 (C_7_); 148.5 (C_4’_); 143.7 (C_3’_); 142.9 (C_8a_); 127.03 (C_2_); 124.6 (C_5_); 120.4 (C_1’_); 119.8 (C_6’_); 116.4 (C_3_); 111.8 (C_2’_ and C_5’_); 111.7 (C_6_), 55.46 and 55.38 (OCH_3_); 52.1 (CH_2_).

#### 2-(4’-Nitrophenyl)-3-hydroxymethyl imidazo[1,2-a]pyridine 6g

Isolated as a yellow solid in 70% yield, mp 225 - 226ºC (Lit 15, mp 224 - 226°C).

IR υ max cm^-1^: 3417 (OH); 3130, 2943 (C-H); 1600 (C = C).

^1^ H NMR (300 MHz, DMSO-d_6_) δ: 8.5 (1 H, d, *J*_5,6_ = 6.9); 8.49 (2 H, d, *J*_2’,3’_ = 8.9); 8.14 (2 H, d, *J*_2’,3’_ = 8.9); 7.62 (1 H, d, *J*_7,8_ = 9.0); 7.34 (1 H, ddd, *J*_5,7_ = 1.2, *J*_6,7_ = 6.9, *J*_7,8_ = 9.0); 7.0 (1 H, ddd, *J*_6,8_ = 1.2, *J*_5,6_ = 6.9, *J*_6,7_ = 6.9); 5.55 (1 H, t, *J* = 5.1, OH); 4.98 (2 H, d, *J* = 5.1, CH_2_).

^13^C NMR (125MHz, DMSO-d_6_) δ: 146.3 (C_5_); 144.2 (C_8a_); 140.8 (C_1_); 140.3 (C_3_); 128.6 (C_3’_); 125.4 (C_4’_); 125.0 (C_2_); 123.4 (C_2’_); 122.2 (C_7_); 116.8 (C_8_); 112.3 (C_6_); 52.0 (CH_2_).

#### 2-Phenyl-3-hydroxymethyl imidazo[1,2-a]pyrimidine 7a

Isolated as a yellow solid in 60.6% yield, mp 241 - 242ºC.

IR υ max cm^-1^: 3216 (OH); 3082, 2948 y 2884 (C-H); 1614 (C=C).

^1^H NMR (300MHz, DMSO-d_6_) δ: 8.92 (1H, dd, *J*_5,6_=6.9, *J*_5,7_=1.2, H_5_); 7.84 (2H, d, *J*_2’,3’_=6.9, H_2’,6’_); 7.61 (1H, dd, *J*_7,6_=8.1, *J*_7,5_=1.2, H_7_); 7.53 (2H, dd, *J*_3’,2’_=6.9, *J*_3’,4’_=7.2, H_3’,5’_); 7.4 (1H, dd, *J*_4’,3’_=7.2, H_4’_); 7.1 (1H, td, *J*_6,5_=6.9, *J*_6,7_=6.9, *J*_6,8_=1.2, H_6_); 5.45 (1H, s, OH); 4.96 (2H, s, CH_2_).

^13^C NMR (75.5MHz, DMSO-d_6_) δ: 150.5 (C_5_), 147.2 (C_8a_); 143.6 (C_1’_), 133.8 (C_2’_); 128.6 (C_3’_); 128.3 (C_4’_); 128 (C_7_); 119.2 (C_3_); 108.6 (C_8_); 111.9 (C_6_); 51.8 (CH_2_).

Anal. calcd for C_13_H_11_N_3_O: C, 69.33 H, 4.88, N, 18.76; found: C, 69.41, H, 5.2, N, 18.46.

#### 2-(4’-Chlorophenyl)-3-hydroxymethyl imidazo[1,2-a]pyrimidine 7b

Isolated as a yellow solid in 63.8% yield, mp 164 - 165ºC.

IR υ max cm^-1^: 3400 - 1900 (OH and C-H); 1662 and 1618 (C=C).

^1^H NMR (500MHz, DMSO-d_6_) δ: 8.97 (1H, d, *J*_5,6_=7.0, H_5_); 8.61 (1H, dd, J_7,6_=4.0, *J*_7,5_=2.0, H_7_); 7.91 (2H, d, *J*_2’,3’_=8.5, H_2’,6’_); 7.58 (2H, dd, *J*_3’,2’_=8.5, H_3’,5’_); 7.15 (1H, dd, *J*_6,5_=7.0, *J*_6,7_= 4.0, H_6_); 5.65 (1H, s, OH); 4.92 (2H, s, CH_2_).

^13^C- NMR (125MHz, DMSO-d_6_) δ: 150.6 (C_7_); 147.2 (C_8a_); 142.2 (C_2_); 133.6 (C_5_); 132.8 (C_1’_); 132.6 (C_4’_); 129.9 (C_2’_); 128.7 (C_3’_); 119.4 (C_6_); 108.7 (C_3_); 56.0 (CH_2_).

Anal. calcd for C_13_H_10_N_3_OCl: 60.11 H, 3.85, N, 16.19; found: C, 60.23, H, 4.01, N, 15.92.

#### 2-(4’-Fluorophenyl)-3-hydroxymethyl imidazo[1,2-a]pyrimidine 7c

Isolated as a yellow solid in 30.3% yield, mp 232 - 233ºC.

IR υ max cm^-1^: 3300 (OH); 3076 (C-H); 1659 and 1615 (C=C).

^1^H NMR (500MHz, DMSO-d_6_) δ: 9.01 (1H, dd, *J*_5,6_=6.5, *J*_5,7_=2.0, H_5_); 8.59 (1H, dd, *J*_7,6_=4.0, *J*_7,5_=2.0, H_7_); 7.92 (2H, dd, *J*_2’,3’_=12.0, *J*_2’F_=5.0, H_2’_); 7.34 (2H, dd, *J*_2’,3’_=12.0, *J*_3’F_=9.0, H_3’_); 7.14 (1H, dd, *J*_6,7_=4.0, *J*_6,5_=6.5 H_6_); 5.72 (1H, s, OH); 4.9 (2H, s, CH_2_).

^13^C NMR (125MHz, DMSO-d_6_) δ: 163.4 and 161.0 (C_4_); 150.4 (C_7_); 147.0 (C_8a_); 142.4 (C_2_); 133.6 (C_5_); 130.2 (C_2’_); 130.1 (C_1’_); 119.2 (C_6_); 115.6 and 115.4 (C_3’_); 108.6 (C_3_); 51.5 (CH_2_).

Anal. calcd for C_13_H_10_N_3_OF: 64.19 H, 4.11, N, 17.28; found: C, 65.26, H, 4.23, N, 16.98.

#### 2-(4’-Methylphenyl)-3-hydroxymethyl imidazo[1,2-a]pyrimidine 7d

Isolated as a yellow solid in 32% yield, mp 199 - 200ºC.

IR υ max cm^-1^: 3369 (OH); 2926 (C-H); 1617 (C=C).

^1^H NMR (500MHz, DMSO-d_6_) δ: 8.99 (1H, dd, *J*_5,6_=6.5, *J*_5,7_=1.5, H_5_); 8.59 (1H, dd, *J*_7,6_=7.0, *J*_7,5_=1.5, H_7_); 7.9 (2H, d, *J*_2’,3’_=8.0, H_2’,6’_) 7.57 (2H, d, *J*_3’,2’_=8.0, H_3’,5’_); 7.13 (1H, dd, *J*_6,7_=7.0, *J*_6,5_=6.5, H_6_); 5.7 (1H, s, OH); 4.91 (2H, s, CH_2_); 3.38 (3H, s, CH_3_).

^13^C NMR (125MHz, DMSO-d_6_) δ: 150.7 (C_7_); 147.1 (C_8a_); 142.2 (C_2_); 133.6 (C_5_); 132.7 (C_1’_); 132.7 (C_4’_); 129.8 (C_3’_); 128.6 (C_2’_); 119.6 (C_6_); 108.6 (C_3_); 55.9 (CH_2_); 51.5 (CH_3_).

Anal. calcd for C_14_H_13_N_3_O: 70.29 H, 5.39, N, 17.57; found: C, 70.13, H, 5.51, N, 17.38.

#### 2-(4’-Methoxyphenyl)-3-hydroxymethyl imidazo[1,2-a]pyrimidine 7e

Isolated as a yellow solid in 86% yield, mp 187 - 188ºC.

IR υ max cm^-1^: 3409 (OH); aprox 2900 (C-H); 1617 (C=C).

^1^H NMR (500MHz, DMSO-d_6_) δ: 8.95 (1H, dd, *J*_5,6_=6.5, *J*_5,7_=2.0, H_5_); 8.56 (1H, dd, *J*_7,6_=4.0, *J*_7,5_=2.0, H_7_) 7.82 (2H, d, *J*_2’,3’_=8.5, H_2’,6’_); 7.12 (1H, dd, *J*_6,5_=6.5, *J*_6,7_=4.0, H_6_); 7.08 (2H, dd, *J*_3’,2’_=8.5, H_3’,5’_); 5.62 (1H, s, OH); 4.9 (2H, s, CH_2_); 3.83 (3H, s, OCH_3_).

^13^C NMR (125MHz, DMSO-d_6_) δ: 159.2 (C_4’_); 149.9 (C_7_); 147.1 (C_8a_); 143.6 (C_2_); 133.3 (C_5_); 129.5 (C_2’_); 118.4 (C_6_); 114.1 (C_3’_); 108.3 (C_3_); 55.2 (CH_2_) 51.7 (OCH_3_).

Anal. calcd for C_14_H_13_N_3_O_2_: 65.88 H, 5.09, N, 16.47; found: C, 65.66, H, 4.80, N, 16.29.

#### 2-(3’,4’-Dimethoxyphenyl)-3-hydroxymethyl imidazo[1,2-a]pyrimidine 7f

Isolated as a brown solid in 30.4% yield, mp 200 - 201ºC.

IR υ max cm^-1^: 3397 (OH); 3083, 2994 and 2937 (C-H); 1615 (C=C).

^1^H NMR (500MHz, DMSO-d_6_) δ: 8.97 (1H, d, *J*_5,6_=4.0, H_5_); 8.56 (1H, d, *J*_7,6_=2.0, H_7_); 7.49 (1H, d, *J*_2’,6’_=2.0, H_2’_); 7.42 (1H, d, *J*_6’,5’_=8.0, H_6’_); 7.09 (2H, m, H_5’,6_); 5.46 (1H, s, OH); 4.93 (2H, s, CH_2_); 3.8 (6H, s, OCH_3_).

^13^C NMR (125MHz, DMSO-d_6_) δ: 149.9 (C_7_); 148.8 (C_4’_); 148.7 (C_3’_); 147.0 (C_8a_); 143.7 (C_2_); 133.3 (C_5_); 126.4 (C_1’_); 120.7 (C_6’_); 118.6 (C_3_); 111.8 (C_2’_ and C_5’_); 108.6 (C_6_), 55.5 and 55.4 (OCH_3_); 51.7 (CH_2_).

Anal. calcd for C_15_H_15_N_3_O_3_: 63.15 H, 5.26, N, 14.73; found: C, 63.01, H, 5.17, N, 14.44.

#### 2-(4’-Nitrophenyl)-3-hydroxymethyl imidazo[1,2-a]pyrimidine 7g

Isolated as a yellow solid in 72% yield, mp 213 - 214ºC.

IR υ max cm^-1^: 3376 (OH); 2933 (C-H); 1675 (C=C).

^1^H NMR (300MHz, DMSO-d_6_) δ: 8.64 (1H, t, *J*_5,6_=6.9, *J*_5,7_=1.2, H_5_); 8.34 (2H, dd, *J*_2’,3’_=8.7, H_3’_); 8.15 (2H, d, *J*_2’,3’_=8.7, H_2’_); 7.1 (1H, dd, *J*_6,8_=1.2, *J*_7,8_=9.0, H_8_); 6.15 (1H, dd, *J*_5,7_=1.2, *J*_6,7_=6.6, *J*_7,8_=9.0, H_7_); 5.59 (1H, d, *J*_6,8_=1.2, H_8_); 4.9 (1H, t, *J*_5,7_=1.2, *J*_6,7_=6.6, *J*_7,8_=9.0, H_7_); 3.32 (1H, t, *J*_5,6_=6.9, *J*_6,7_=6.6, *J*_6,8_=1.2, H_6_); 2.9 (1H, d, *J*=5.1, OH); 2.4 (2H, d, *J*=5.1, CH_2_).

^13^C NMR (125MHz, DMSO-d_6_) δ: 152.7 (C_5_); 148.6 (C_8a_); 148.06 (C_1_); 142.3 (C_3_); 141.6 (C_3’_); 135.2 (C_4’_); 125.0 (C_2_); 130.3 (C_2’_); 125.2 (C_7_); 122.6 (C_8_); 110.3 (C_6_); 52.88 (CH_2_).

Anal. calcd for C_13_H_10_N_4_O_3_: 57.77 H, 3.70, N, 20.74; found: C, 57.56, H, 3.85, N, 20.62.

## Conclusion

2-Aryl-3-hydroxymethyl imidazo[1,2-a]pyridines and 2-aryl-3-hydroxymethyl imidazo[1,2-a]pyrimidines emitted light at long wavelengths more intensely than the respective non-substituted imidazo[1,2-a]pyridine and pyrimidine fluorophores. The hydroxymethyl group was an enhancer of the fluorescence intensity of the 2-aryl imidazo[1,2-a]azines, although in some cases it caused a decrement of the luminescent activity. These findings indicate that an interplay of the electronic character of the phenyl substituents with the hydroxymethyl moiety is most probably operating. The thermogravimetric analysis on selected most fluorescent imidazo[1,2-a]azines, indicated that they are thermally stable up to 280°C. Other studies aimed to decide if the herein investigated compounds are suitable as effective fluorescent dyes in biological applications are currently underway.

## Competing interests

The authors declare that they do not have competing interests.

## Authors’ contributions

MVP observed high fluorescence activity when preparing 3-hydroxymethyl derivatives by reaction of the imidazo[1,2-a]pyridine with formaldehyde in an acidic media. HSZ proposed and designed the project. SVO confirmed the observation made by MVP, carried out the synthesis and characterization of all new compounds, and participated in both the qualitative and quantitative photophysical analysis. CPG, ARA and ECA contributed in the analysis of all spectral data and discussed with HSZ the course of the investigation. All authors read, made comments and approve the final manuscript.
